# Right Hydronephrosis as a Complication of Acute Appendicitis

**DOI:** 10.1155/2016/3231862

**Published:** 2016-03-16

**Authors:** Selahattin Koray Okur, Yavuz Savaş Koca, İhsan Yıldız, İbrahim Barut

**Affiliations:** Department of General Surgery, Suleyman Demirel University Medical Faculty, 32260 Isparta, Turkey

## Abstract

*Introduction*. Acute appendicitis is the most common cause of acute abdomen, but atypical appendicitis may lead to delayed diagnosis and related complications. In this report, we present a very rare case of acute appendicitis causing right hydronephrosis.* Case Report*. A 54-year-old male patient who had been receiving antibiotic therapy due to the diagnosis of urinary tract infection for the last one week but had no clinical improvement was admitted to the emergency service. Abdominal computed tomography (CT) showed right hydronephrosis and a pelvic abscess. After appendectomy and abscess drainage had been performed, hydronephrosis was completely recovered.* Discussion*. The use of appendicitis scoring systems, abdominal ultrasonography (USG), abdominal CT, and diagnostic laparoscopy can be useful for the diagnostic process in patients presenting with acute abdomen. In our patient, we considered that the surgical treatment was delayed since the symptoms of acute appendicitis were suppressed by the antibiotic therapy that was being administered due to the complaints including symptoms of urinary tract infections.* Conclusion*. Atypical appendicitis may cause a delay in the diagnosis of acute appendicitis and thus may lead to serious complications such as right hydronephrosis, prolonged hospital stay, increased morbidity and mortality, and increased antibiotic resistance.

## 1. Introduction

Acute appendicitis is the most common cause of acute abdomen and can be seen in any age group [1]. Typical cases present with paraumbilical pain migrating to the right lower quadrant of the abdomen, leading to rebound tenderness in the right lower quadrant. Moreover, typical, uncomplicated cases are easy to diagnose. However, the diagnosis of atypical appendicitis is a difficult task and thus delayed diagnosis may lead to perforation. If the perforation is limited by the surrounding organs, particularly by the small bowel and omentum, localized peritonitis with plastron or periappendiceal abscess occurs. On the other hand, if the perforation is not limited, generalized peritonitis is seen. The complications caused by delayed diagnosis lead to increased morbidity and mortality [2].

In this study, we present a patient who had hydronephrosis with the compression of the right ureter as a complication of acute appendicitis, which is a very rare condition in the literature.

## 2. Case Report

**Figure 1:**
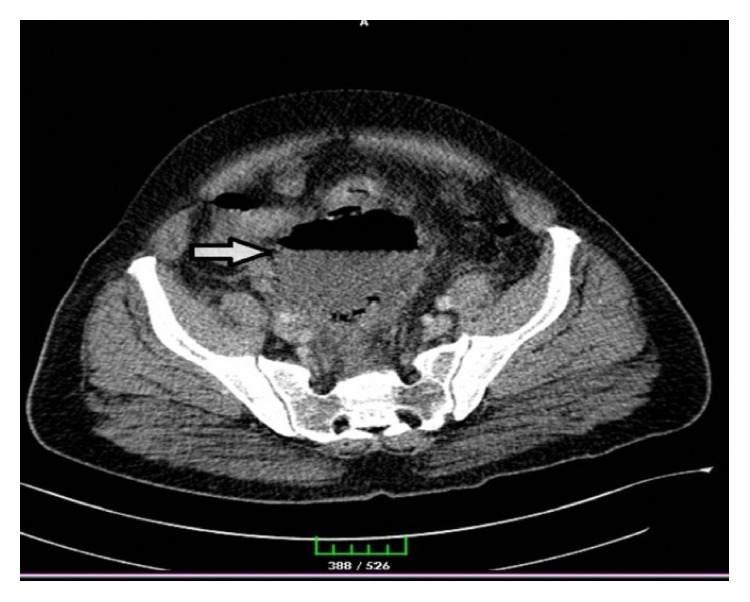
CT image showing 98 × 95 mm abscess with air-fluid levels at the entrance to the pelvic cavity.

**Figure 2:**
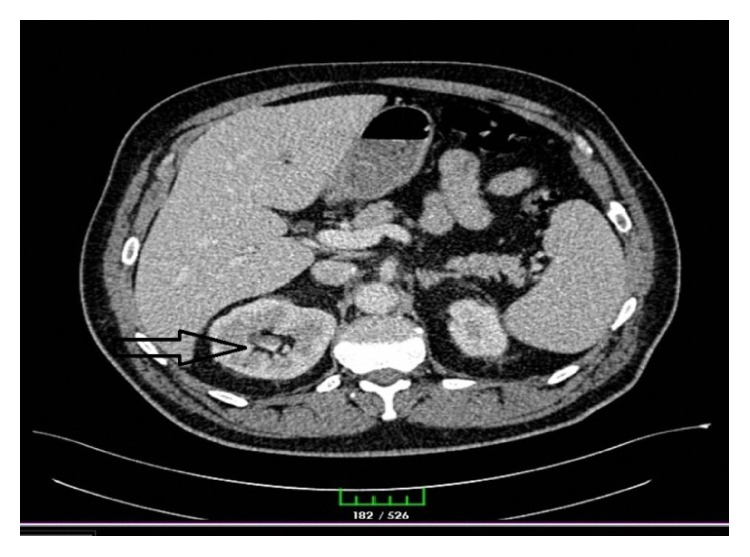
CT image showing hydronephrosis in the right kidney.

A 54-year-old male patient had been diagnosed with urinary infection and had been on antibiotic therapy for the last one week due to the complaints of right flank pain, suprapubic pain, constipation, nausea, vomiting, and inappetence and was admitted to the emergency service due to the absence of clinical relief of symptoms. Physical examination revealed abdominal defence and rebound tenderness in the suprapubic region. The patient had no temperature, and no pathology was detected in the rectal examination. Laboratory workup revealed leukocyte 18,600/*μ*L, neutrophil 88.3%, C-reactive protein (CRP) (nephelometric) 223 mg/L, fasting glucose 191 mg/dL, creatinine (blood) 1.83 mg/dL, and BUN 34 mg/dL as well as blood (+) and leukocyte (+) in urine analyses. A computed tomography (CT) scan showed a 98 × 95 mm abscess in the midline pelvis with air-fluid levels and hydronephrosis in the right kidney (Figures 1 and 2). Abdominal ultrasound (USG) showed nonvisualized appendix, right hydronephrosis, dilatation in the right ureter, and diffuse inflammation in the pelvis. Depending on these signs and symptoms, the patient was operated on under emergency conditions. Surgical exploration showed a perforated appendix and an abscess particularly on the right side and also extending towards the posterior sigmoid colon. Appendectomy, abscess drainage, and intraperitoneal irrigation were performed; a drain was inserted in the right quadrant and another drain in the pelvic cavity. The patient was given a combined therapy of intravenous metronidazole 0.5% and ceftriaxone 1 gr 2 × 1 in the following days. Follow-up USG revealed complete recovery of hydronephrosis. At postoperative day 7, laboratory workup revealed leukocyte 11,400/*μ*L, neutrophil 66.3%, C-reactive protein (CRP) (nephelometric) 51.5 mg/L, fasting glucose 103 mg/dL, creatinine (blood) 1.22 mg/dL, and BUN 27 mg/dL as well as blood (−) and leukocyte (−) in urine analyses. The drains were removed, the treatment was switched to oral antibiotics, and the patient was discharged with no complications. Pathological analysis indicated acute perforated appendicitis.

## 3. Discussion

Atypical appendicitis leads to delayed diagnosis, complications, and an increased risk of morbidity and mortality. Atypical appendicitis is mostly seen in patients aged under five or over 65 years. Pregnant patients may also have an atypical presentation due to displacement of the appendix. Moreover, atypical appendicitis may be caused by inflammatory diseases such as Crohn's disease, use of steroids or immunosuppressant drugs, and use of antibiotics prior to diagnosis [3]. Similarly, our patient was also receiving antibiotics during hospital admission.

The growing use of scoring systems such as the Alvarado Score and the Appendicitis Inflammatory Response Score, which include various items such as periumbilical pain migrating to the right lower quadrant, inappetence, nausea or vomiting, rebound tenderness in the right iliac fossa or muscular defence, temperature, leukocytosis, and the presence of neutrophils showing a left shift and elevated CRP elevation, has led to more accurate diagnosis of acute appendicitis [4]. In our patient, the Alvarado Score was 4 and the Appendicitis Inflammatory Score was 7. These scores indicated that diagnostic tests, active follow-up, and diagnostic laparoscopy were needed.

Abdominal CT is a golden-standard diagnostic tool in the patients with a pain in the right lower quadrant since it reduces the incidence of unnecessary surgery and treatment costs [3]. Although CT provides more accurate findings when compared to USG, the latter is the method of choice in children, pregnant patients, and the patients with radiation exposure since it is cost-effective and simple and, more importantly, does not use ionizing radiation [5]. In our patient, the diagnosis was established by CT since the patient was considered to have a complicated condition due to the presence of atypical appendicitis, elevated leukocyte level, neutrophil dominance, and increased CRP level.

Laparoscopic appendectomy has been shown to be more commonly performed when compared to open appendectomy since laparoscopy reduces the risk of incisional hernia, can be used in the differential diagnosis of suspected appendicitis, potentially decreases postoperative pain and length of hospital stay, and enables the patients to quickly return to normal activity [6]. Nevertheless, laparoscopic appendectomy leads to a greater risk of intra-abdominal abscess [6]. To avoid perforation-induced complications, the acceptable negative appendectomy rate may be 15% in patients with suspected appendicitis [1]. Recently, a number of studies have reported that several patients with uncomplicated appendicitis have been treated and followed up by antibiotic therapy. It has also been reported that spontaneous recovery may occur in patients with symptoms of appendicitis [7]. However, nonoperative treatment may lead to failure in 9% of the patients and may result in postoperative complications and perforation in half of the patients. Delay in surgical intervention caused by failure of conservative treatment may lead to prolonged hospital stay and increased morbidity, and the delayed diagnosis of an underlying cancer in the appendix or cecum may result in antibiotic resistance [8].

Appendicitis can mimic urinary system symptoms, mainly because of its proximity to the right ureter. In particular, compression of the right ureter may mimic urinary tract infections and may lead to urinary obstruction as well. Ureteral compression with hydronephrosis is a rare complication of acute appendicitis. Moreover, bilateral urinary obstruction may also be seen, though rarely [3, 5, 9].

In conclusion, atypical appendicitis may cause a delay in the diagnosis of acute appendicitis, leading to complications and prolonged hospital stays. Therefore, in patients presenting with an abdominal pain, acute appendicitis should be kept in mind, emergency surgery should be performed following the definitive diagnosis, and the scoring systems, imaging techniques, and, if needed, diagnostic laparoscopy should be performed in patients with suspected appendicitis.
